# Investigating the role of peptides in effective therapies against cancer

**DOI:** 10.1186/s12935-022-02553-7

**Published:** 2022-03-27

**Authors:** Reza Naeimi, Asrin Bahmani, Saeid Afshar

**Affiliations:** 1 Research Center for Molecular Medicine, Hamadan University of Medical Sciences, Hamadan, Iran; 2 Department of Molecular Medicine, School of Advanced Medical Sciences and Technologies, Hamadan University of Medical Sciences, Hamadan, Iran; 3 Department of Medicinal Plants and Natural Products Research Center, Hamadan University of Medical Sciences, Hamadan, Iran

**Keywords:** Peptides, Cancer, Therapeutic effect, Vaccines

## Abstract

Early diagnosis and effective treatment of cancer are challenging. To diagnose and treat cancer effectively and to overcome these challenges, fundamental innovations in traditional diagnosis and therapy are necessary. Peptides can be very helpful in this regard due to their potential and diversity. To enhance the therapeutic potential of peptides, their limitations must be properly identified and their structures engineered and modified for higher efficiency. Promoting the bioavailability and stability of peptides is one of the main concerns. Peptides can also be effective in different areas of targeting, alone or with the help of other therapeutic agents. There has been a lot of research in this area, and the potential for variability of peptides will continue to improve this process. Another promising area in which peptides can help treat cancer is peptide vaccines, which are undergoing promising research, and high throughput technologies can lead to fundamental changes in this area. Peptides have been effective in almost all areas of cancer treatment, and some have even gone through clinical phases. However, many barriers need to be overcome to reach the desired point. The purpose of this review is to evaluate the mechanisms associated with peptides in the diagnosis and treatment of cancer. Therefore, related studies in this area will be discussed.

## Introduction

Despite many advances in cancer treatment and detection, the management of disease remains one of the most challenging areas. Many researchers are studying both the diagnosis and treatment of cancer, and their main concern in cancer diagnosis is the early detection of cancer. In this case, patients can be kept hopeful about treatment, and even the side effects of treatment, which are another concern, can be reduced. In the field of treatment, the most important concerns have been increasing the bioavailability of therapeutic agents and reducing the side effects of therapies [1, 2]. In most parts of the world, most conventional chemotherapies are prescribed systemically, and after a while, due to unpleasant side effects such as systemic manifestations or drug resistance, they are stopped, and in most cases, there are no suitable alternatives [3, 4].

A variety of molecules have been developed to target therapy, some of which are mentioned here. Aptamers are one of the most effective molecules in the field of targeting. Despite their benefits in the field of targeting, their main challenges are sensitivity and degradation in the face of enzyme-containing tumor environments. Monoclonal antibodies (mAbs) are effective in treating and targeting cancerous tissues. Some of them have even been able to enter the market. But despite these achievements, one of the major issues with their use is their large size, which makes it difficult for them to penetrate tumor tissue, although the high cost of their production can also limit their use [5–8]. However, many studies have proven the promising potential of peptides in the treatment of various cancers. In general, peptides that have been researched in the field of cancer treatments have been prepared from various sources, the most important of which have been based on natural sources, the production of recombinant peptides, and the synthesis of peptides in the laboratory [9, 10]. Anticancer therapies based on these peptides have used more than two specific strategies, are: (A) These peptides have been used as mimics of natural peptides that have been involved in cancer-related signaling pathways, (B) These peptides have the potential to pass through the membrane and lead to the delivery of therapeutic agents. So they have been studied and used in the field of therapeutic agent delivery [11]. To succeed in the laboratory and clinical phases and to confirm the widespread use of peptides as beneficial therapeutic agents, there are several challenges that researchers seek to overcome. The most important of these challenges are the variety of exopeptidases and endopeptidases in the physiological environment that therapeutic peptides must protect against before they reach the tumor environment and even after they reach the tumor environment. [12–14]. Peptides also have low bioavailability, which is addressed by various solutions, especially in the field of nanotechnology [15]. Another obstacle is early clearance of peptides, and nanocarriers have been designed to ensure the stable release of therapeutic peptides [16–18]. Finally, most peptides enter the body by intravenous injection. One of the major challenges is the instability in serum that must be overcome [19].

Due to the extensive research related to therapeutic peptides in the field of cancer, this review attempts to provide information on types of cell-penetrating peptides (CPPs), peptides conjugated in nanostructures (such as liposomes, cationic polymer nanoparticles, etc.), peptides developed in targeting, and finally, peptide vaccines to be categorized, and to provide examples of successful research in each of these areas. Although attempts have been made to categorize the order of research studies based on their importance and efficiency, due to the breadth of the content, the order of the content is not exactly related to their importance, and the taste and interest of authors have played a role in choosing the order. In this review, we will focus on the role of peptides in reducing therapeutic concerns.

### Engineered peptides to increase cellular uptake and further stability

Several efficient peptides have been developed from animal and plant sources for the treatment of various cancers. However, most of these peptides are restricted in terms of stability, targeting, and bioavailability [18, 20]. Although several strategies have been proposed to optimize these peptides. Cycling peptides, L-amino acids substitution with D-amino acids, and synthesizing hybrid peptides to target therapeutic goals are some of the strategies that are discussed below [21, 22]. Peptide engineering is the process of changing the structure or communication capacities of peptides such that they have higher stability or cellular uptake than their natural form. It is therefore a unique phenomenon in the field of cancer treatment.

One of the most effective peptides in cancer treatment is pro-apoptotic peptides. In fact, one of the carcinogenic mechanisms is disruption of normal cell apoptosis, which will eventually lead to malignancy, and even an increase in anti-apoptotic agents in drug resistance has been proven. Pro-apoptotic peptides have apoptosis induction functions in cancer cells, but a main problem with the use of these peptides is their limited entry into target cells [18]. To overcome this problem, appropriate delivery systems have been designed that have increased the uptake of these peptides. The use of hybrid nanoparticles and peptides are examples of these efficient delivery systems. Akrami et al. evaluated increasing the permeability of the alpha-lipoic acid-peptide conjugate, LAWKRAKLAK, through surface modifications of gold nanoparticles [23]. Jung et al. achieved interesting results in developing a hybrid peptide. The results of this study showed that they succeeded in inducing apoptosis in bladder cancer cells through the Bld-1-KLA peptide. KLA causes apoptosis in HT1376 cells, whereas Bld-1 targets this peptide [24].

l-amino acids are used in the structure of human proteins and peptides. By replacing some of these amino acids, d-peptides have been synthesized that contain d amino acids in their structure. These peptides are resistant to proteases and do not elicit unwanted immune responses. However, these peptides also have limitations, the most important of which is their low uptake by target cells. Nanocarriers are used to improve this problem. This method is being extensively researched and has had promising results [25]. Zhou et al. synthesized a d-tetrapeptide that could remain phosphate for the tyrosine residues and effectively inhibiting cancer cells that overexpressed alkaline phosphatase [26].

One of the most effective ways to increase the stability of peptides is the synthesis of cyclic peptides. These peptides are resistant to exonucleases due to the lack of N-terminals and C-terminals, and it has also been found that their circular shape and specific conformation are more efficient in terms of Gibbs free energy for bonding with targets [27]. Modifications to one of the epidermal growth factor receptor (EGFR) domains and cyclic peptide production, for example, were used to create a cytotoxic state for cancer cells [28].

### Peptides for active and passive targeting of tumors

**Fig. 1:**
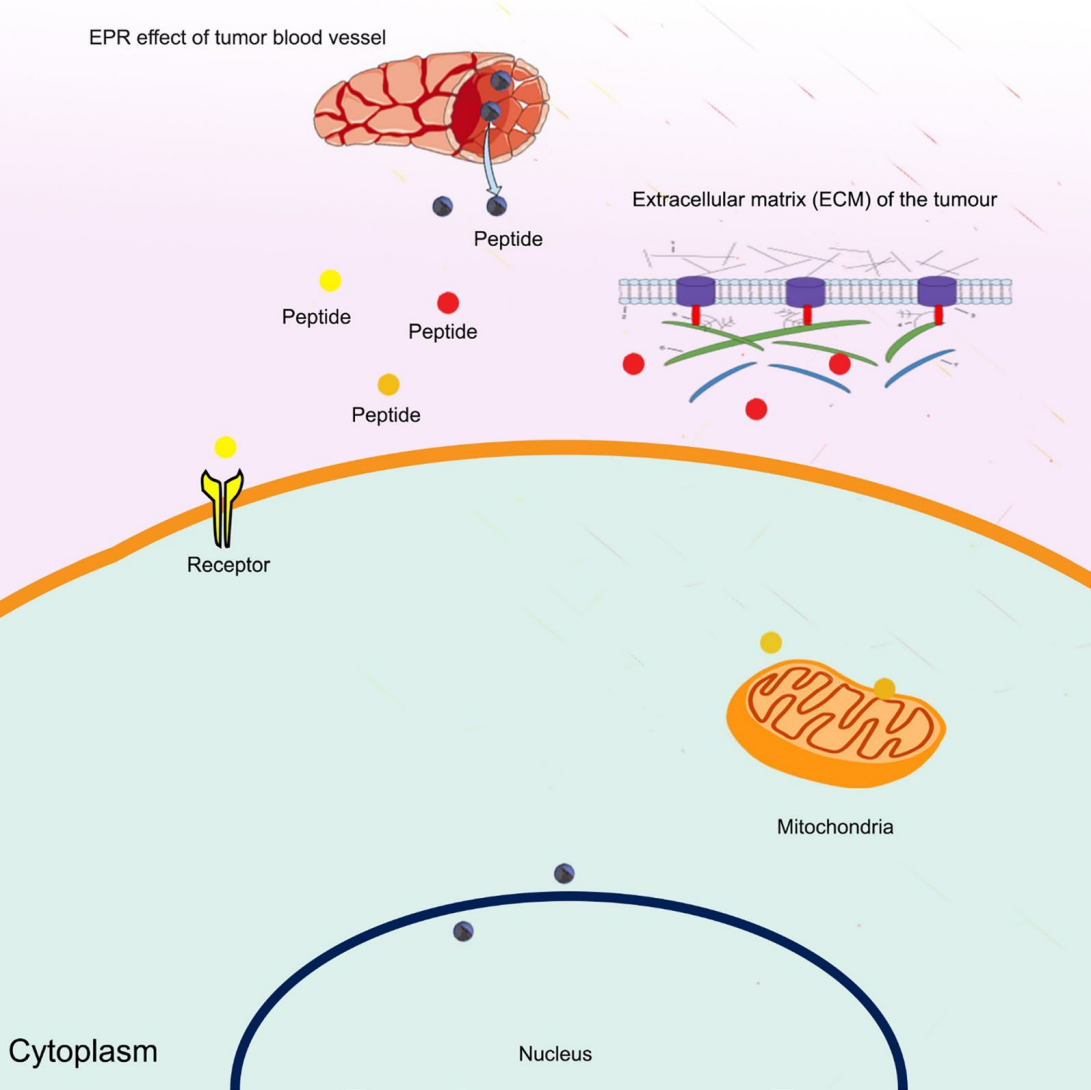
Application of peptides in the field of active and passive targeting. Tumor tissue has certain properties that can be used for target treatment, and peptides are a good choice for targeted treatment due to their small size and potential for manipulation and engineering. Their small size will be effective in the enhanced permeability and retention (EPR) effect (passive targeting), and different peptides can bind to different cell surface receptors or be used against targets in the extracellular matrix (ECM) or in intracellular organelles

Some receptors on the surface of tumor cells increase, which can help treat many cancers more effectively. In fact, molecules such as peptides can target these receptors on the surface of tumor cells, so-called “active targeting therapy”. In addition, tumor tissues, and especially the vessels around the tumor tissue, have features such as abnormal endothelium that allow therapeutic agents to be placed in them, so-called passive targeting therapy. Peptides can play a role in both of these targeted therapies. For this purpose, researchers engineer them to increase the tendency to specific receptors or, in terms of size or tendency, to specific tumor microenvironments (Fig. 1). In general, some peptides have the ability to target cell surface receptors. Some peptides can be effective in targeting the microenvironment around tumors (e.g., extracellular matrix targeting), and some peptides can even cross plasma membranes and target intracellular organelles such as mitochondria [29]. Table 1 summarizes the peptides that have proven to be effective in targeting.

**Table 1:** Targeting peptides that studied in the treatment of cancers

Peptide	Cancer	Cellular target	Outcomes	References
M-CTX-Fc	Glioblastoma	CD44	Increased bioavailability of DOX in U251MG-P1 glioblastoma cells. It was carried by liposomes	[30]
TAT	Nasopharyngeal carcinoma	CPP	Reduce the drug resistance and side effects of cisplatin	[34]
BR2	Cervical cancer, melanoma and colon cancer	CPP	Antiproliferative effects on HeLa, HCT116 and B16-F10 cells	[35]
cRGD	TNBC	αvβ3 integrin	Suppressed tumor growth with no noticeable side effects	[46]
cRGD	Lung	αvβ3 integrin	Increased bioavailability and anticancer effects of Dox	[47]
CTL-1	Glioma	Fibronectins	Increased inhibitive effects of PTX	[49]
MRP	Liver cancer	MMP-2	Increased antifibrotic effects of Pirfenidone	[50]
MTP-NRP1	Glioblastoma	NRP-1	Decreased cancer tissue angiogenesis due to this intervention	[51, 52]
SP2043	Breast cancer	IGF1R	Biomimetic peptide inhibits growth and metastasis of breast cancer by inhibiting the lymphangiogenesis and angiogenesis	[53]
TAT-ELP1-L12	Pancreatic adenocarcinoma	CPP	Enhance the antitumor effects of L12 peptide through dual targeting	[62]
GLVATVKEAGRSIHEIPREEL	Lymphoma	BCL6	Maintain the corepressor activity of BCL6 and prevent the unbridled increase of B cells	[72]
TAT-ETD	Hepatocarcinoma	CPP	HIF-1 activity and expression level reduction under hypoxia and finally reduced tumor growth by inhibiting angiogenesis	[73]

One of the deadliest brain cancers is glioblastoma. Doxorubicin (DOX), which has been shown to be effective in the treatment of that, to increase the bioavailability of this drug, various target therapies have been investigated, including the research of Mahmoud et al., They inserted Dox into the liposomes and targeted the liposomes with M-CTX-Fc (chlorotoxin peptide fused to human IgG Fc region without hinge sequence) for U251MG-P1 glioblastoma cells. In these cells, CD44 receptors are overexpressed, and Peptide M-CTX-Fc is designed to target these receptors [30].

Cell-penetrating peptides (CPPs) are other peptides that are effective in penetrating tumor tissue. They have a short sequence of 5–30 amino acids and cross the membrane with or without energy consumption [31]. Due to this permeability, they are known as Trojan peptides. Tumor tissues are poorly perfused due to abnormal vascular and epithelium, and therefore permeability factors such as these peptides can increase the effectiveness of treatment [32]. In the structure of these peptides, both positively charged and negatively charged amino acids can be present. The existence of positively charged amino acids in these peptides causes an electrostatic interaction with the negative charge of cell membrane phosphates. This electrostatic interaction facilitates cell entry for CPP peptides. On the other hand, negatively charged amino acids can be important in terms of local aggregations [33]. The first known cationic CPP was the transactivator of transcription (TAT) peptide, which was discovered from the HIV virus [5]. Wang et al. studied reduce the drug resistance and side effects of cisplatin by using TAT peptide. According to their method, iron oxide nanoparticles were used. In this study, the peptide was able to mediate the targeted delivery of the drug by this nanoparticle [34]. BR2 peptide was also developed by Lim et al., which had antiproliferative effects on three cell lines: HCT116 cells, HeLa cells, and B16-F10 cells. The therapeutic effects of this peptide are mediated by interactions with cancer cell gangliosides. [35].

Mitochondria, as the most important cellular organelle in the field of cell energy supply, also play a very important role in apoptosis, and because it contains circular DNA with genomic DNA-independent genes, it can mutate some important genes. In this regard, it is involved in cancer progression, especially in the metastatic stage. Due to the importance of mitochondria in the progression of cancer, peptide-based therapies have been established to target mitochondria, which usually contain KLA peptides. In this treatment method, in order to improve the treatment process, drugs such as Dox are also targeted [36]. One of the challenges in treating all types of cancer is the presence of cancer stem cells, which often cause the recurrence of the disease. Cancer stem cells have been shown to have more mitochondria than other stem cells, so targeting the mitochondria of these cells can be an effective treatment [37, 38]. A type of metallopeptide (MPP) that binds to dichloro (1,10-phenanthroline) copper (II) synthesized by Laws et al. could induce cell death in breast cancer stem cells. By stimulating mitochondrial membrane depolarization and reducing cytochrome C activity, MPP is able to selectively kill cancer stem cells. In addition, it was found that MPP has been involved in increasing the JNK and P38 signaling pathways as well as increasing reactive oxygen species (ROS) within the mentioned stem cells [39].

Many cancer-related therapeutic agents affect the expression of genes within the nucleus. However, entry into the nucleus requires certain signals. This includes the nuclear localization sequences (NLS) that allow therapeutic peptides to enter the nucleus [40]. This ability of peptides to target has recently been used to target some drugs. For example, an effective hybrid is composed of platinum and NLS peptide (Pt-NLS) that can effectively enter the nucleus. [41]. The exosome is one of the best nanocarriers for therapeutic and diagnostic agents, which has recently received a lot of attention from researchers, and because of its innate nature, it can have very promising achievements. Cheng et al. evaluated a photosensitizer along with a chimeric protein containing an NLS peptide through an exosomal vector for targeted therapy, which improved anti-tumor efficacy and enhanced ROS effect [42]. It has been shown that TAT peptides and NLS sequences can be used simultaneously for dual targeting and efficiency enhancement. By Leung et al., this method was used to target Nanodiamonds (NDs) for gene therapy via oligonucleotide ANA4625. In addition to therapeutic effects, this nanostructure is also used in imaging [43].

RGD motifs can target the ECM of tumor tissues. For example, DM1, a potent inhibitor of tubulin polymerization, can be used as a (HER2)-positive metastatic breast cancer treatment. Zhong et al. used the cRGD peptide to target DM1 against TNBC (a highly heterogeneous subtype of breast cancer) [44–46]. In TNBC, αvβ3 integrins are overexpressed and are suitable for targeting. Lim et al. used micelles carrying Dox via RGD peptide to target this integrin in U87MG cancer cells [47]. In addition to integrins, fibronectins (FNs) and matrix metalloproteinases (MMPs) are attractive targets that have been studied in the ECM. Fibronectins are abundant in the extracellular matrix and are overexpressed in some cancers. By Zhang et al. CTL-1 protein was used to target glioblastoma cancer FNs by PEG-PLA nanoparticles containing Paclitaxel (PTX) [48, 49]. The MRP protein is sensitive to MMP-2 and has been effectively evaluated in research. Pirfenidone has been shown to have antifibrotic effects and can increase the permeability of the extracellular matrix. Ji et al. used liposomes containing Pirfenidone were targeted by protein MRP and used against pancreatic cancer tissue. The findings of this study revealed that as the ECM becomes more permeable to gemcitabine, the drug's therapeutic benefits improve [50].

One of the causes of cancer progression is abnormal angiogenesis, which can be exacerbated by some factors. For example, in the fatal cancer of glioblastoma, glycoprotein NRP-1 has been shown to increase angiogenesis. By Nasarre et al. a peptide MTP-NRP1 (Transmembrane domain sequence NRP1) was evaluated to improve treatment and had promising results due to its effective antiangiogenic effects [51, 52]. Also, SP2043 peptide (derived from collagen IV), which can block IGF1R, has been shown to have good antiangiogenic effects [53].

Another advantage of peptides is that they can respond to internal and external stimuli. Therefore, peptides can be targeted against cancer cells based on these stimuli. For example, peptides have been engineered to respond to different pH, pressures, and enzymes [54]. The Warburg effect, which has shown that cancer cell metabolism is mostly anaerobic based on glycolysis and fermentation, is one of the hallmarks of cancer cells. For this reason, the environment around cancer cells has a low pH. So this is important in terms of targeting [55]. PHLIP Peptides are a family of bacteriorhodopsin peptides that have been considered in both imaging and drug delivery. This group of peptides, in response to low pH, protonates and enters the cell membrane [56, 57]. In order to improve targeting and increase the rate of penetration into tumor tissue, this peptide has been engineered by modifying effective amino acids in response to pH [58]. The TH peptide is also an effective pH-responsive peptide that is positively charged at low pHs, such as the pH around cancer cells. By Xia et al. this peptide was used to target liposomes containing the two drugs PTX and losartan against breast cancer cells. The results of this study showed that concomitant use of this peptide and losartan increases the accumulation of PTX in breast tumors. Although losartan is used as an antihypertensive drug, it has been shown to be effective in preventing the dense collagen network around the tumor. As the results of this study show, its concomitant use in chemotherapy can increase the bioavailability of the anticancer agent at the tumor site, and it has been shown that losartan does not have antihypertensive effects in this type of targeted treatment. [59–61]. Some peptides are also engineered to respond to temperature, which can make the treatment process more targeted. For example, in an interesting study, TAT-ELP1-L12 peptide was synthesized and evaluated against pancreatic adenocarcinoma. In chimeric peptides, TAT peptide acts as a CPP, while ELP1 peptide and L12 (derivative of bovine lactoferrin) peptides act as temperature responders and therapeutic agents, respectively [62]. Chimeric peptide Bac-ELP-H1 has been synthesized with a similar structure in terms of the temperature response component. This peptide has been developed to treat brain-related cancers. In response to hyperthermia, the percentage of penetration of this peptide into the cell increases, and after a temperature pulse, this peptide again becomes soluble. As a result of these two modes, a concentration gradient is created for the peptide, which contributes to the greater permeability of the peptide [63].

In terms of targeting, the use of bacteriophages as carriers of diagnostic and therapeutic agents is helpful. These viruses can be targeted through the phage display technique or decorated with pathogen epitopes that mimic PAMPs (pathogen-associated molecular patterns). Phages also have the ability to stimulate the innate immune system and adaptive immune system with these changes [64, 65]. Shadidi et al. employed M13-based 7- and 12-mer commercial phage libraries, and it was determined that two breast cancer-specific internalizing peptides (LTVSPWY and WNLPWYYSVSPT) were shown to deliver fluorescein-conjugated anti-Her2 antisense oligonucleotides into SKBR3 cells [66]. Cai et al. study indicated that a plasmid coding for a siRNA against focal adhesion kinase (FAK) was easily packed into M13 particles displaying epithelial growth factor (EGF) as a fusion with the gp3 protein. They then used this phage against H1299 lung carcinoma cells, and the results showed that this structure reduced cell growth and invasion [67].

One of the most effective methods of targeted treatment based on peptides is peptide-drug conjugates (PDCs). In this method, the therapeutic agent is attached to a peptide via a linker. The peptide acts as a homing device in this structure [68]. Lu 177 dotatate is currently the only PDC that has been able to successfully pass all clinical phases and enter the market. This PDC is currently used to treat gastroenteropancreatic neuroendocrine tumors and is FDA approved [69]. There are two promising types of PDCs, including bicycle-toxin conjugates and peptide-dendrimer conjugates. Various clinical trials for these PDCs are currently underway [68]. For example, BT1718, BT5528, and BT8009 are bicycle-toxin conjugates that are being evaluated for targeting tumors in different phases [70]. Peptide-dendrimer conjugates are being studied in the preclinical phase and have promising prospects for entering the clinical phases of cancer treatment [68].

Dysfunction of transcription factors (TFs) that control the expression of genes can lead to a variety of diseases, including cancer, impaired response to hormones, and impaired growth [71]. Targeting transcription factors is a vast and fascinating field that will be the subject of much cancer research. Peptides, especially peptides that interact with proteins, are of particular importance in this regard, examples of which are discussed below.

The GLVATVKEAGRSIHEIPREEL peptide has been produced as a synthetic ligand for BCL6 (an important transcription factor in multiple pathways), and the results suggest that it is an effective treatment for cancers such as lymphoma. BCL6 normally acts as a corepressor in B cells, but in the pathway leading to cancer, one of its natural ligands disrupts its corepressor function, and if this trend continues, it will lead to the proliferation of B cells and cancer [72]. HIFs are important transcription factors that are directly controlled by the mTOR signaling pathway, and if they have increased expression, the induction of angiogenesis will increase. In addition, these TFs have been shown to be involved in the migration and metastasis of cancer cells. There is an ERK Targeted Domain (ETD) in HIF-1α that has been shown to mutate variants of this domain that reduce HIF-1α activity. Therefore, as a treatment method, variants of ETD have been developed. By Karagiota et al. one of the variants of ETD was fused to a TAT peptide. The results showed that TAT-ETD peptides reduced the activity of HIF-1α. Also found that hypoxia caused the death of cancer cells following a decrease in HIF-1α activity [73].

### Peptide vaccines

Vaccines have been around for a long time to prevent or reduce the severity of infectious diseases, but there is still a long way to go to prevent many cancers with vaccines. To create a strong immune response, the immune system must be stimulated against tumor-associated antigens (TAAs) [74]. TAAs are usually recombinant proteins or synthetic peptides, although it has been shown that antigen-specific T cell responses can be prompted by synthetic peptides [75]. Despite the high hopes for this type of vaccination, promising clinical findings have yet to emerge [76]. It is believed that this failure was due to the inadequate immunogenicity of these vaccines, and it has been found that in most cases, due to a lack of optimization, the formulation, adjuvants, and even the route of administration of these vaccines should be changed [77–79].

Peptide vaccines generally contain epitopes for TAA-specific T cells. Apart from peptides, various types of antigens can be used to activate the immune system. However, synthetic peptides have advantages over them that have expanded their use. Among other things, synthetic peptides are easily produced and more cost-effective than recombinant proteins. Their cost-effectiveness is more related to not needing a GLP cell-processing center [80]. In addition, peptide vaccines are more flexible against antigen/epitope loss than antibodies, and this feature in peptide vaccines is due to the use of multiple epitopes and the possibility of changing the peptides for different epitopes [81, 82]. Finally, stimulation of a specific TCR can be considered one of the benefits of individual peptides. This advantage is due to the fact that CD8 + T cells (CTLs) are able to recognize irrelevant antigens that are commonly found in large proteins, engineered viruses, or tumor cell-based vaccines. These will create harmful immune responses. But, these nonspecific responses occur less frequently due to the presence of more specific epitopes in peptides [83].

**Fig. 2:**
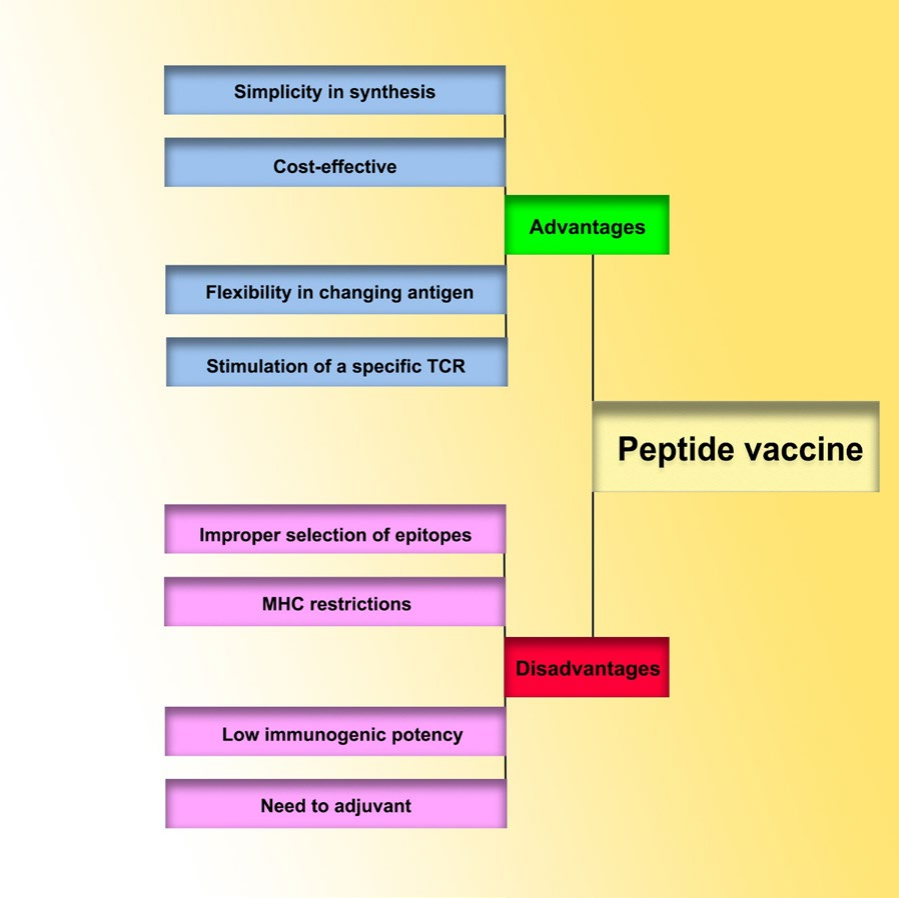
Advantages and disadvantages of peptide vaccines for cancer. Although peptide vaccines have advantages such as ease of synthesis, cost-effectiveness, flexibility to antigens, and high specificity, they also have disadvantages such as false positive and negative responses, MHC restrictions, low immunogenic potency, and the need for adjuvant that are being addressed by various studies, and some of these studies have even entered clinical trials

The disadvantages of peptide vaccines include false-positive and false-negative responses, which require full knowledge of effective MHCs and epitopes to prevent them [84, 85]. The extent and diversity of MHC alleles in different populations and races also challenge the widespread availability of peptide vaccines. [86]. In addition, it should be noted that peptide vaccines have lower immunogenic potency than proteins, and adjuvant can be used to overcome this inefficiency. Some cancers also show low levels of MHC1, which in this case would make it challenging to use peptide vaccines unless these vaccines are used in combination with other treatments that increase the level of MHC. For example, peptide vaccines can be used in combination with targeted therapies against EGFR [86]. In general, peptide vaccines have extensible advantages and compensable disadvantages (Fig. 2), so it can be hoped that these vaccines will have broader applications in the not-too-distant future.

In the preclinical and clinical phases, a series of peptides for the construction of anticancer vaccines have shown promising outcomes, which are briefly discussed. Peptides have been extracted from Bcl-2, Bcl-XL, and Mcl-1 proteins and are being evaluated as a multiepitope vaccine in phase I clinical trial [87]. Peptides from HER-2 receptor epitopes have been conjugated with liposomes and have shown good results in preclinical studies [88]. The MUC1 tandem repeat peptide has been assessed in the structure of numerous peptide vaccines, and it is now being studied for various vaccines in phases I to III of clinical trials after showing promising results in preclinical investigations [89]. Peptide Hp91, derived from the HMGB1 cytokine, has been evaluated as an adjuvant in the HPV vaccine, which has been shown to induce increased secretion of interferon and antibodies compared to other adjuvants [90]. The NSC 635 089 peptide has been evaluated as an adjuvant in cancer vaccines and has been shown to activate natural killer cells and raise antibody levels. Studies have shown that it has a cytotoxic effect on Dalton’s lymphoma ascites (DLA) cells and prevents the growth of tumor cells. In this regard, this peptide is both a vaccine and a therapeutic agent [91].

In the end, what looks promising for the production of peptide anti-cancer vaccines is the human advancement in high-throughput sequencing (HTS) technologies. These technologies can detect mutations that result in the appearance of antigens on the surface of cancer cells called neo-antigens. These antigens have unique properties that stimulate the immune system based on them, reducing risks such as autoimmune diseases. Accurate identification of these antigens, as well as the development of vaccines based on them, could be very promising [92, 93].

## Conclusion

The most important concerns for various cancer treatments are the therapeutic side effects of chemotherapy and drug resistance that may occur at different steps of treatment. Therefore, the need for new treatment strategies is inevitable, and peptides are considered one of the best therapeutic agents for cancer. Peptides have unique and proportionate properties, such as small size and simplicity of synthesis. There are major challenges to using peptides as targeted therapeutic agents, such as disproportionate physical and chemical properties that must be changed in line with therapeutic goals as well as peptides' limited affinity for cellular targets, which necessitates peptide structural alterations.

One of the obstacles to the development of studies up to the clinical stage is the different conditions of conducting experiments in the preclinical and clinical trial stages, which lead to results that, in some cases, have serious contradictions. It appears that offering more precise protocols and correctness in publishing articles without publication bias is of special value in increasing study speed and overcoming this obstacle. Also, one of the technologies that can help the rapid development of peptide-based therapies is nanotechnology, which can be effective in reducing toxicity and improving the targeting of therapeutic peptides. In addition, the conjugation of peptides with nanoparticles can be a less invasive route for the administration of peptides. Currently, peptides are mostly administered through the parenteral route, which has its own problems.

## Data Availability

Not applicable.

## Abbreviations

mAbs: Monoclonal antibodies
CPPs: Cell-penetrating peptides
EGFR: Epidermal growth factor receptor
DOX: Doxorubicin
M-CTX-Fc: Chlorotoxin peptide fused to human IgG Fc region without hinge sequence
CPPs: Cell-penetrating peptides
TAT: Transactivator of transcription
MPP: Metallopeptide
ROS: Reactive oxygen species
NLS: Nuclear localization sequences
Pt-NLS: Platinum and NLS peptide
NDs: Nanodiamonds
FNs: Fibronectins
MMPs: Matrix metalloproteins
PTX: Paclitaxel
HTS: High throughput sequencing
